# A Comparative Analysis of Laser-Ablated Surface Characteristics Between the Si Face and C Face of Silicon Carbide Substrates

**DOI:** 10.3390/mi16010062

**Published:** 2025-01-01

**Authors:** Hsin-Yi Tsai, Yu-Hsuan Lin, Kuo-Cheng Huang, J. Andrew Yeh, Yi Yang, Chien-Fang Ding

**Affiliations:** 1 Taiwan Instrument Research Institute, National Applied Research Laboratories, Hsinchu 300092, Taiwan; kellytsai@narlabs.org.tw (H.-Y.T.); marklin@narlabs.org.tw (Y.-H.L.); 2 Department of Power Mechanical Engineering, National Tsing Hua University, Hsinchu 300044, Taiwan; jayeh@mx.nthu.edu.tw; 3 Department of Biomechatronics Engineering, National Taiwan University, Taipei 106319, Taiwan; yanne90313@gmail.com (Y.Y.); cfding@ntu.edu.tw (C.-F.D.)

**Keywords:** silicon carbide (SiC), ultraviolet laser, hardness, surface roughness, oxidation

## Abstract

Silicon carbide (SiC) has significant potential as a third-generation semiconductor material due to its exceptional thermal and electronic properties, yet its high hardness and brittleness make processing costly and complex. This study introduces ultraviolet laser ablation as a method for direct SiC material removal, investigating the effects of varying scanning speeds on surface composition, hardness, and ablation depth. The results indicate optimal processing speeds for the Si and C faces at 200 mm/s and 100 mm/s, respectively. Ablation depth is linearly correlated with laser repetitions, achieving a 25% improvement in removal efficiency at 100 mm/s on the C face compared to higher speeds. A composition analysis shows that the Si and C faces of SiC exhibit consistent ratios of Si, O, and C both before and after ablation. Post-ablation, the proportion of Si and C decreases with an increased presence of oxygen. At scanning speeds below 200 mm/s, the variation in speed has minimal effect on the compositional ratios, indicating a stable elemental distribution across the surface despite differences in processing speed. Hardness testing indicates an initial hardness of 13,896 MPa for the C face, higher than that of the Si face, with both surfaces experiencing a drop to less than 1% of their original hardness (below 50 MPa) after ablation. Lattice structure analysis shows Moissanite-5H SiC and cubic silicon formation on the Si face, while the C face retains partial SiC structure. This study found that when laser parameters are used to process SiC, the processing parameters required on both sides are different and provide important reference information for future industrial processing applications to shorten the time and process cost of SiC surface thinning.

## 1. Introduction

Silicon carbide (SiC) is an advanced semiconductor material, primarily due to its superior electrical and thermal properties compared to traditional silicon (Si), and has become one of the key materials in the third-generation semiconductor industry [1,2]. SiC possesses a wide bandgap, enabling it to function efficiently at higher temperatures, voltages, and frequencies, making it ideal for high-power, high-temperature applications such as electric vehicles, renewable energy systems, and industrial power electronics [3,4,5]. Additionally, SiC’s ability to withstand a higher breakdown of electric fields allows for smaller, more efficient power devices, thereby enabling miniaturization and reducing overall system costs. Furthermore, SiC’s high thermal conductivity and low thermal expansion can reduce energy losses and improve heat dissipation, which significantly enhances device reliability and lifespan. Despite its numerous advantages, SiC presents several challenges in the semiconductor industry, primarily due to the complexity of its processing and its high production costs. The material’s high hardness contributes to its durability, but also makes it difficult to machine and polish, resulting in longer manufacturing times and the need for specialized equipment. Additionally, the growth of large, defect-free SiC crystals is both technologically demanding and costly, further increasing the overall manufacturing expenses. These factors contribute to higher production costs compared to traditional Si substrates.

After SiC is sliced using a wire saw, the wafer surface typically exhibits significant surface damage, including deep saw marks and subsurface cracks due to the mechanical stress imparted during sawing. The grinding process is the first step in minimizing these imperfections. Grinding progressively removes the surface damage and reduces the wafer’s thickness to the desired specification. After grinding, polishing is conducted to further reduce surface roughness and remove any residual defects, such as fine scratches or shallow cracks left by grinding. The polishing process for SiC usually involves chemical-mechanical polishing (CMP), where abrasive particles in a slurry work in combination with chemical agents to achieve material removal at the atomic level. Polishing SiC is particularly difficult due to the material’s chemical inertness and hardness, which makes it resistant to both mechanical and chemical interactions. The slow material removal rate (MRR) in the CMP process for SiC leads to longer processing times, and controlling the uniformity of the polished surface is challenging. Zhou et al. [6] explored the improvement results of CMP technology in SiC wafers by introducing catalyst nanoparticles into the slurry. This method significantly improves MRR and produces defect-free, ultra-smooth surfaces on Si-side SiC wafers. It was found that using Fe and Pt/C nanoparticles as catalysts can improve the oxidation efficiency during polishing. Especially for Fe catalysts, SiC wafers achieve atomically flat, defect-free surfaces and extremely low Ra of about 0.05 nm. They are proven to be more effective in increasing MRR compared to traditional methods, making CMP processes more efficient in industrial applications. Additionally, to improve the speed and efficiency of the grinding and polishing processes for silicon carbide (SiC), several advanced methods and auxiliary processes were employed to enhance the material removal rates, reduce process times, and maintain surface integrity. Hsieh et al. [7] introduced the key advancement in CMP for SiC. There are some major challenges in achieving high MRR while maintaining a smooth surface and minimal subsurface damage due to its hardness and the different removal rates of the Si and C sides. Therefore, the hybrid CMP technologies were introduced and described in the study, which included electro-CMP (ECMP), Fenton-ECMP, ultrasonic-ECMP, photocatalytic CMP (PCMP), and gas-PCMP. These techniques aim to increase the oxidation efficiency of SiC, facilitating faster material removal while enhancing surface quality.

Lasers are widely utilized in the field of material processing, particularly for surface treatments, cutting, drilling, and texturing. In surface treatment applications, lasers change the material characteristics by altering surface textures, improving or reducing hardness, or changing surface roughness according to its advantages of high energy density, direct writing, and precision control. In the CMP process, the laser system can be induced to enhance polishing efficiency by thermally softening the material’s surface layer, thereby accelerating the material removal rate (MRR). This approach is particularly beneficial for hard materials like silicon carbide (SiC) and sapphire. The thermal effects of laser assistance reduce the mechanical force required, leading to decreased tool wear, minimized surface damage, and improved surface finish. The above results in a more efficient CMP process, enhancing overall throughput and surface quality in semiconductor applications. Therein, laser-assisted CMP on SiC surfaces use different laser types, such as picosecond (ps), femtosecond (fs) [8,9], and nanosecond (ns) lasers [10,11], each offering distinct advantages and disadvantages. Picosecond and femtosecond lasers can create ultra-short pulses that limit heat diffusion, enabling precise ablation with minimal thermal damage and surface stress. However, their high cost and complexity may limit widespread use. In contrast, nanosecond lasers are cost-effective and more accessible but generate a larger heat-affected zone, which can lead to surface damage and residual stress. Therefore, the selection of appropriate laser types and the fine-tuning of laser parameters are crucial for softening SiC surfaces to facilitate CMP. Key laser parameters, such as pulse duration, wavelength, and energy density directly affect the thermal and ablation properties, enabling controlled material removal and minimizing subsurface damage. Gao et al. [12] investigated using picosecond laser pretreatment (PL) to enhance CMP on the Si face of 6H-SiC wafers. The results indicated that the ripple structures and polycrystalline layer generated by PLP enhance the machinability of the SiC surface. The formation of C-O, Si-C-O, and Si-O bonds during PLP facilitates material removal in the CMP process. Consequently, compared to non-laser pretreated (NLP) samples, PLP-treated samples exhibit a significantly higher MRR during the initial 45 min of CMP, along with lower surface roughness (Rq) following CMP. Xie et al. [13] used the femtosecond laser to irradiate the SiC substrate and discuss its influence on the CMP process. The control parameters of the laser system include the laser fluence, focus position, scanning speed, and scanning interval on the surface of SiC. The study also discussed the influences of the surrounding conditions on the surface morphology of laser-processed SiC. The results revealed that Si–O compounds formed on laser-irradiated SiC surfaces, which are advantageous for the subsequent CMP process due to their relatively low hardness. Zhang et al. [14] explored the femtosecond laser polishing of SiC ceramics, evaluating the effects of pulse energy and defocus on surface morphology, roughness, depth, and oxidation. The results revealed that at a 175 kHz repetition rate, 1064 nm wavelength, and 9-s ablation time, the laser ablation threshold for SiC is 0.355 J/cm². As the pulse energy increases, the surface roughness first decreases and then increases, and the polishing depth generally increases. Larger defocus reduces energy density, resulting in reduced ablation capability, reduced polishing depth, and increased roughness. Finally, the degree of oxidation is relatively unaffected by changes in pulse energy and defocus. Zhao et al. [15] investigated the femtosecond laser ablation of 4H–SiC wafers, analyzing how laser pulse energy, defocus, repetition rate, and scan intervals affect ablation depth, width, and surface morphology. The results showed that the laser formed a U-shaped microgroove at a 6 mm defocus. By optimizing the scanning interval, an ideal surface roughness (Sa) of 0.267 μm can be achieved, and brittle features such as microcracks and pits on the original surface can be effectively removed. The process achieves surface modifications that facilitate subsequent polishing processes, like CMP, by creating smoother surfaces and enabling easier material removal. Wang et al. [16] discussed various femtosecond laser processing techniques, including direct laser processing, composite processing, environmental modifications, and beam shaping, each tailored for SiC’s unique properties. By applying the femtosecond laser processing to SiC, it can achieve precise material removal and modifications at micro and nanoscale levels. Wang et al. [17] presented a novel nanosecond laser irradiation pretreatment to enhance the CMP of single crystal SiC by irradiating the Si face before CMP and using a 1 wt% diamond+ 99 wt% deionized water eco-friendly slurry in the CMP process. Experimental results show that nanosecond irradiation improves surface quality and optimizes microstructure and chemical composition, which helps optimize MRR and surface roughness. An et al. [18] addressed the processing challenges of RB-SiC composites by using nanosecond laser irradiation to soften the material. The process resulted in a maximum hardness reduction of 40.7%, and the degree of softening is highly correlated with laser parameters. Chemical composition analysis showed that the SiC content decreased, and the Si content increased after laser irradiation, which was the main reason for the decrease in hardness of RB-SiC composite materials. This work gave a feasible route to improve the removability of the RB-SiC composite.

The aforementioned research indicates that, while some studies have employed nanosecond lasers for the surface modification of SiC and investigated their removal rates in the CMP process, the majority of research remains focused on the effects of femtosecond lasers on SiC surfaces. Additionally, the Si face and C face of SiC differ in several key aspects, affecting their suitability for various applications. Structurally, the Si face is terminated with silicon atoms, while the C face is terminated with carbon atoms [19], resulting in distinct surface polarities that influence growth rates, doping, and etching behavior. The Si face exhibits a smoother, more uniform surface and generally higher surface quality after polishing, while the C face tends to have higher hardness and may display faster etching rates [20], beneficial in some chemical processes. Due to the aforementioned differences in characteristics, the Si face and C face of SiC exhibit variations in MRR and post-polishing surface roughness during CMP. Consequently, numerous studies [21,22] have focused on the incorporation of different polishing slurries to enhance both the MRR and surface roughness of SiC.

The above-mentioned research focuses on adding polishing slurries with different components to CMP to improve the MRR of SiC and using a laser to pretreat SiC to analyze its impact on properties such as hardness and surface roughness. However, direct dry laser ablation of the Si and C faces of SiC, specifically examining laser parameter effects on each surface’s characteristics, has yet to be fully explored. Therefore, in this paper, we propose ultraviolet nanosecond laser dry ablation of the Si face and C faces of SiC. By analyzing the effects of laser scan speed and repetition rate on removal depth, hardness, composition, and lattice structure, we establish optimal ablation and material removal parameters for each face. This method, a non-contact, consumable-free material removal process, offers a rapid optical direct-write technology for dry thinning of SiC, reducing the reliance on wet grinding processes and the use of consumables like grinding heads and polishing slurry. This method can be applied to the SiC wafer manufacturing industry, greatly reducing process time and cost, thereby expanding the application fields of SiC.

## 2. Fundamental Theory

**Figure 1:**
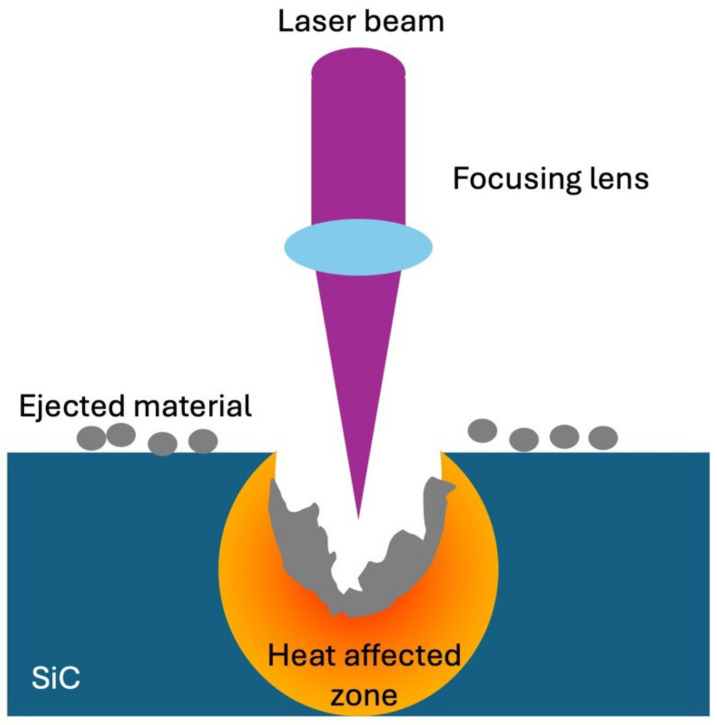
Schematic of a laser ablation on the SiC substrate.

Nanosecond laser ablation is a promising approach for the dry material removal of SiC, primarily due to its ability to generate high-intensity, short-duration pulses that induce rapid localized heating and material ejection. During nanosecond laser ablation, the high photon energy within each pulse interacts with the SiC surface, causing a rapid temperature to rise and subsequent material vaporization or plasma formation [14], as shown in Figure 1. This ablation mechanism effectively reduces material hardness at the target surface, enabling selective layer-by-layer removal without extensive thermal damage to the surrounding areas.

**Figure 2:**
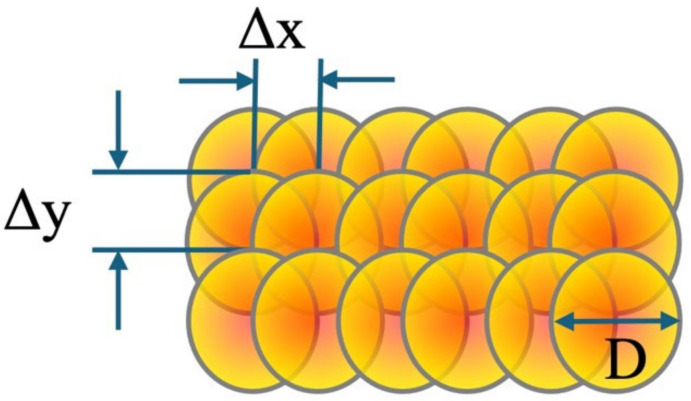
Schematic of the spacing of laser spot and overlap rate in X and Y directions.

The overlap rate of laser spots plays a crucial role in controlling surface morphology and ablation efficiency. A higher laser spot overlap rate promotes a more uniform distribution of energy across the surface, increasing ablation and enhancing material removal depth. Conversely, a lower overlap ratio may cause irregular ablation patterns and leaving untreated. Surface roughness is significantly influenced by the melting and ablation dynamics during material removal. This spot overlap rate can be controlled by adjusting the scanning interval, speed, and pulse repetition frequency. The overlap rate (*O_R_*) can be calculated based on the spot’s bite size (B*_s_*) and diameter (D), as expressed in Equation (1). Moreover, the B*_s_* value could be represented in Equation (2), where the V and F are the scanning speed and pulse repetition frequency, respectively. In the study, the pulse repetition frequency is fixed at 100 kHz, with the overlap in the X direction adjusted through scanning speed, and in the Y direction through scan intervals. In this experiment, the Y direction *O_R_* is fixed at 50%. A schematic of the X and Y directional spot intervals is shown in Figure 2.

(1) $$O_{R}=\frac{D-B_{s}}{D}\times 100\%$$

(2) $$B_{s}=\frac{V}{F}$$

## 3. Materials and Experimental Setup

### 3.1. Materials

Silicon carbide (SiC) is a covalent compound composed of silicon and carbon atoms with the chemical formula SiC. SiC has a variety of crystal structures, called polytypes, the most common of which are 3C, 4H, and 6H [23]. These polytypes vary in their atomic stacking orders and arrangements, resulting in distinct physical and electrical properties. Both 4H-SiC and 6H-SiC are hexagonal crystals, differing in their layer stacking order; 4H-SiC follows an ABCB sequence, while 6H-SiC follows an ABABAB sequence.

Although the lattice arrangement is different, both 4H-SiC and 6H-SiC have the advantages of high hardness, excellent thermal conductivity and chemical resistance. In thermal conductivity, 4H-SiC exhibits higher conductivity along the c-axis, whereas 6H-SiC has superior basal plane conductivity, making it ideal for high-quality substrates. Osipov et al. [24] analyzed the mechanical properties of SiC from the Si and C-faces through nanoindentation techniques. The results showed that the Young’s modulus of the C face almost coincides with the Young’s modulus of bulk 4H-SiC sample, which is approximately 2.3 times higher than the value at Si face. In addition, the hardness at the surface of C face is on average about 1.5 times higher than at the Si-face at a depth of 0–60 nm. (Reviewer#2). In terms of electrical characteristics, 4H-SiC boasts higher electron mobility than 6H-SiC, making it preferable for high-frequency and high-power applications [25]. In industrial applications, the usage rate of 4H-SiC is also higher than that of 6H-SiC. Therefore, 4H-SiC is selected in this study to investigate laser dry ablation effects on its Si and C faces, analyzing the influence of laser parameters on surface characteristics.

### 3.2. Experimental Setup

#### 3.2.1. System and Parameters

**Figure 3:**
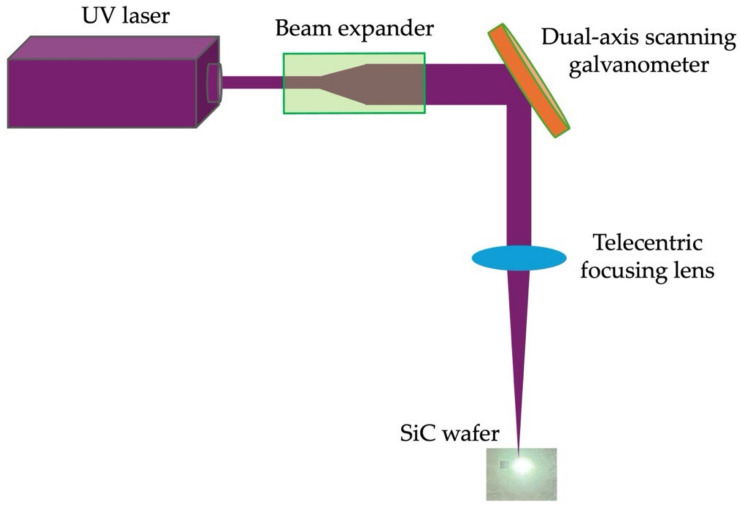
Schematic of the laser dry ablation system on the SiC substrate.

The laser direct dry ablation system includes a solid-state Q-switched ultraviolet laser with a wavelength of 355 nm (Coherent, Inc., Santa Clara, CA, USA, AVIA NX 355-20). The maximum output power of this laser is 20 W. To improve the effectiveness of the laser in SiC dry ablation, this power was used for all experiments in this study. During experiments, the pulse repetition frequency is fixed at 100 kHz, yielding a pulse energy of 200 μJ, the highest pulse energy the laser can produce. The laser is also equipped with a dual-axis scanning galvanometer for two-dimensional scanning over the processing area, and a telecentric focusing lens with a focal length of 580.8 mm, producing a single-point laser spot of approximately 45 μm. The spot interval in the Y direction is set to 22.5 μm, achieving a 50% overlap rate. By adjusting the scanning speed to change the spot overlap rate in the X direction, the scanning speed is set to 50–800 mm/s. Then, the surface removal depth is used to find the better speed range for subsequent surface property analysis such as hardness, composition analysis, and lattice structure. Finally, a better scanning speed was found, and the number of repetitions was varied to explore its effect on surface removal depth. The laser dry ablation setup for SiC is illustrated in Figure 3, and all laser specifications and experimental parameters are summarized in Table 1.

#### 3.2.2. Analysis of Surface Characteristics

In this study, a three-dimensional confocal laser scanning microscopy (KEYENCE Inc., Osaka, Japan, VK-X-2000) is utilized to measure the ablation depth and surface roughness following laser ablation. This technique offers precise, non-contact measurement capabilities that enable high-resolution profiling of surface topography, which is essential for understanding material removal depth and the resulting surface morphology. Using Scanning Electron Microscopy with Energy Dispersive Spectroscopy (SEM-EDS) for compositional analysis before and after laser ablation of SiC enables a detailed examination of elemental changes at the material’s surface. SEM-EDS combines high-resolution imaging with precise elemental identification, allowing us to detect changes in silicon and carbon composition or identify oxidation states that may form during laser processing. This analytical capability facilitates process optimization in applications where specific surface properties or enhanced machinability are required.

For crystallographic analysis, X-ray Diffraction (XRD) is employed to monitor potential lattice structure changes in SiC due to laser processing. XRD is well suited for identifying phase transformations and alterations in crystal orientation, which are crucial for understanding how laser ablation might affect SiC’s material properties. During the measurement, a theta-2theta (θ-2θ) scan was used, with the 2theta angle ranging from 20 to 80 degrees and an increment of 0.08 degrees per step. Finally, nanoindentation testing (Anton Paar Inc., Neuchâtel, Switzerland, NHT^3^) is conducted to assess the hardness of SiC before and post-ablation. This method offers high spatial resolution and sensitivity, enabling precise hardness measurements even in microstructurally complex regions, thereby providing a direct measure of any mechanical property changes due to laser treatment. The above tools offer a comprehensive suite for analyzing SiC’s surface and structural modifications after laser ablation.

#### 3.2.3. Experimental Process

The laser dry ablation process for analyzing the effects of different parameters on the Si face and C face of SiC in terms of surface and material characteristics follows three main steps:

1. Initial ablation parameter setup: place SiC on the preset working plane of the laser system. This position is the focus of the telecentric focusing lens. Then, set the parameters of the laser system. Its power, repetition frequency, and scanning speed are shown in Table 1. Initial high-speed ablation depths are measured to establish baseline values, with subsequent reductions in scan speed. These depths are analyzed to determine whether changes in ablation depth correspond proportionally with speed adjustments, aiming to identify optimal scan speeds for the Si face and C face. Each parameter is tested at least three times, with averaged results used for analysis.
2. Low-speed ablation analysis: due to lower ablation efficiency at high scan speeds, subsequent analyses focus on slower speeds (50–400 mm/s) and assess the effects of laser ablation on both faces of SiC, examining hardness, composition, and lattice structure changes. Detailed methodologies for these analyses are described in Section 3.2.2.
3. Optimal speed and repetition analysis: the optimal speed for effective ablation on the Si and C faces is determined based on experimental results. This speed is then used for repeated ablation passes to achieve a material removal depth target of over 20 μm per face. The number of required passes and the corresponding relationship with material hardness are evaluated and recorded.

## 4. Experimental Results and Discussion

To investigate the influence of laser scanning speed on the surface characteristics of SiC and find the most suitable processing parameters, a UV laser system was used to fix the laser power, pulse frequency, and spot overlap rate in the Y direction, and to adjust the scanning speed to analyze its effect on ablation. Laser power, pulse repetition frequency, and spot overlap rate in the Y direction were held constant, while the scanning speed was varied to examine its effects on ablation depth, surface roughness, and morphology. Subsequently, within an optimized speed range, further analysis will focus on material composition, lattice structure, and hardness, incorporating repeated scans to assess the effects of repetition on ablation depth and hardness. The results of these experiments are detailed in the following sections.

### 4.1. Influence of Scanning Speed on Surface Morphology of SiC

**Figure 4:**
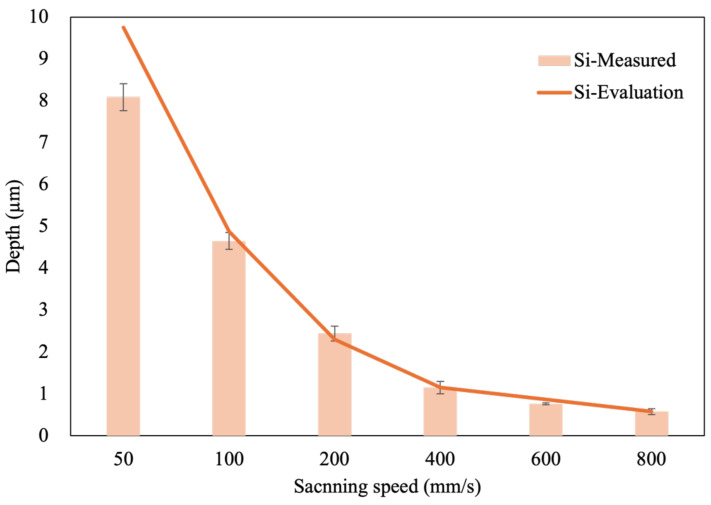
Depth of the Si face of SiC ablated with different scanning speeds.

**Figure 5:**
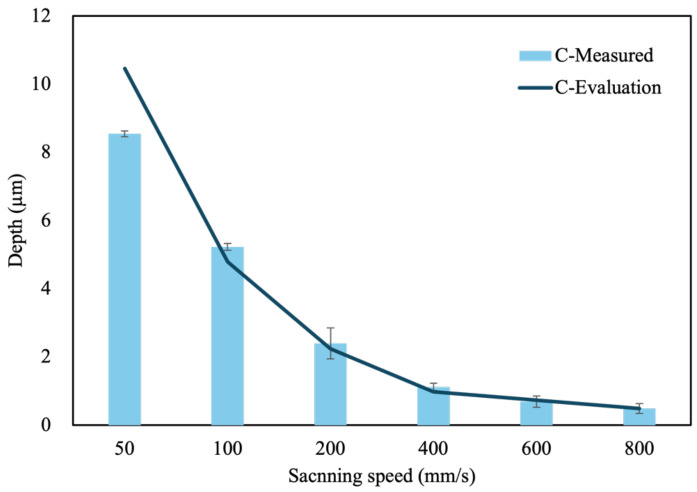
Depth of the C face of SiC ablated with different scanning speeds.

Initially, a scanning speed of 800 mm/s was established as the baseline parameter. The actual ablation depths on the Si and C surfaces were found to be 0.58 μm and 0.49 μm, respectively. By proportionally reducing the scanning speed, the corresponding estimated ablation depths were evaluated, and the actual ablation depths were subsequently measured. The evaluation values are depicted in the curves shown in Figure 4 and Figure 5, while the measured ablation depths are represented in a bar graph.

The experimental results indicate that for the Si surface, the actual ablation depths at scanning speeds of 400 mm/s and 600 mm/s are shallower than the evaluated values. However, at a scanning speed of 200 mm/s, the actual ablation depth exceeds the evaluated value. Further analysis of the ablation depth at a scanning speed of 200 mm/s revealed that when the speed is decreased to 100 mm/s and 50 mm/s, the actual ablation depths again fall below the evaluated values. These results suggest that the optimal scanning speed for the Si surface is 200 mm/s, yielding an average ablation depth of approximately 2.3 μm.

Conversely, on the C surface, the actual ablation depths at scanning speeds ranging from 200 mm/s to 600 mm/s are consistently shallower than the estimated depths derived from a proportional reduction from the 800 mm/s baseline. Only at a scanning speed of 100 mm/s does the actual ablation depth surpass the estimated value. However, when the scanning speed is further reduced to 50 mm/s, the actual ablation depth again fails to meet the estimated value. Therefore, the optimal scanning speed is determined to be 100 mm/s for the C surface.

**Figure 6:**
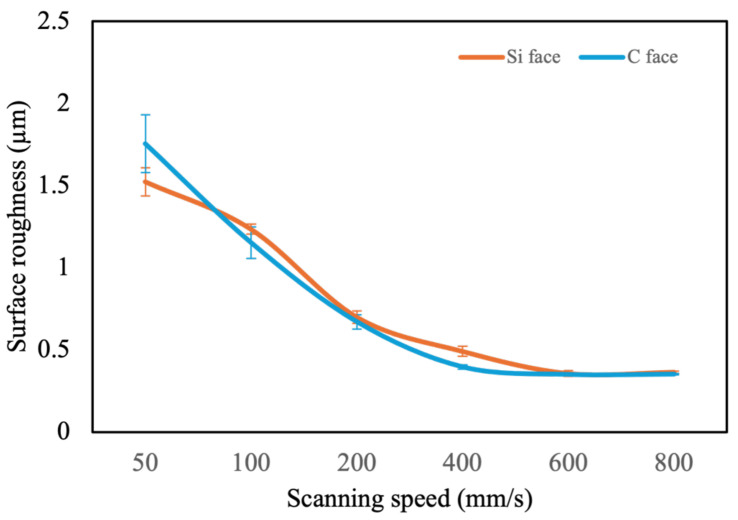
Surface roughness of SiC ablated with different scanning speeds.

**Figure 7:**
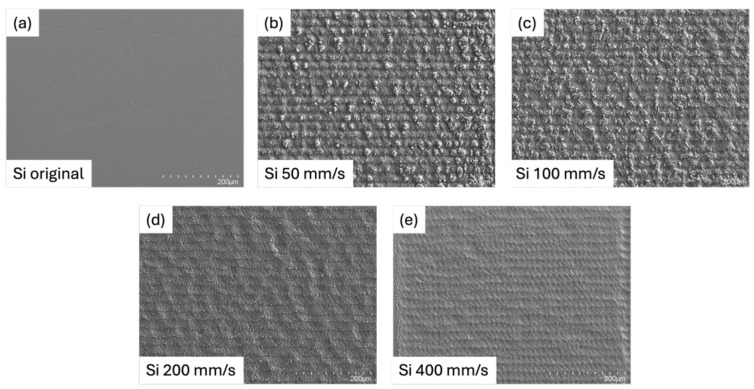
SEM image of the Si face of SiC (**a**) before and ablated with different scanning speeds of (**b**) 50 mm/s, (**c**) 100 mm/s, (**d**) 200 mm/s, and (**e**) 400 mm/s.

The graph shows the relationship between scanning speed and surface roughness for both the Si face and C face of SiC during laser ablation as shown in Figure 6. As scanning speed decreases from 800 mm/s to 50 mm/s, surface roughness increases for both faces, indicating smoother surfaces at higher scanning speeds. At the highest speed of 800 mm/s, both faces achieve minimal roughness, with values consistently below 0.5 μm, indicating smooth surface characteristics. However, as the speed decreases to 400 mm/s and further down to 100 mm/s, surface roughness rises notably, with values reaching above 1.5 μm at the lowest speed of 50 mm/s. Figure 7 shows SEM images of the Si face surface at 200× magnification, comparing surface morphology before and after laser ablation. Across the scanning speed range of 100–800 mm/s, the Si face consistently shows slightly higher surface roughness than the C face. However, at a lower speed of 50 mm/s, this trend reverses, with the C face displaying greater roughness than the Si face. This observation indicates that as the scanning speed decreases, the ablation effect becomes less uniform and results in rougher surfaces on both faces. Furthermore, under reduced scanning speeds, the Si face demonstrates a slightly greater resistance to increases in surface roughness compared to the C face, suggesting a marginally better tolerance to roughness variation in slower processing conditions.

### 4.2. Effect of Laser Ablation on SiC Material Propertiess

The experimental results shown in Figure 4 and Figure 5 reveal that at scanning speeds above 400 mm/s, the ablation depth per pass on both the Si face and C face of SiC is less than 1 μm. This shows that the removal efficiency is not high at high scanning speeds. Consequently, further analysis was conducted to investigate the effects of laser ablation on material properties at lower scanning speeds. Notably, the primary material characteristic affected by laser ablation on SiC is its hardness. The observed change in hardness may be attributed to alterations in lattice structure or oxidation of the ablated material. Therefore, this study explores the differences between the Si face and C face in terms of hardness, elemental composition, and lattice structure after laser ablation.

**Figure 8:**
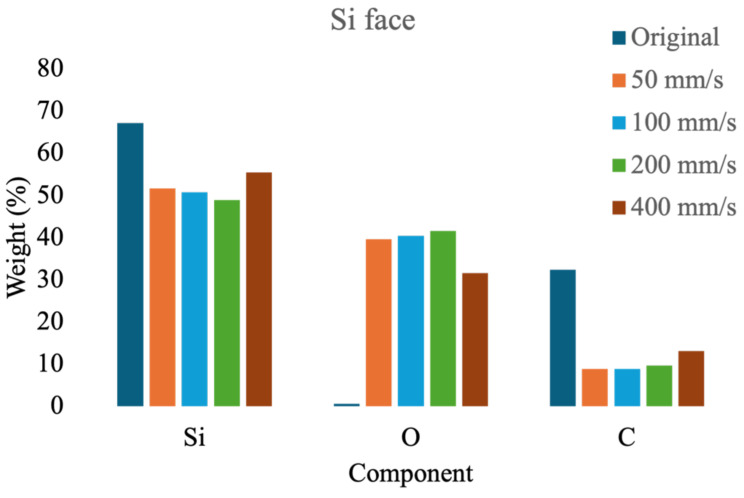
Ingredient analysis of the Si face of SiC ablated with different scanning speeds.

**Figure 9:**
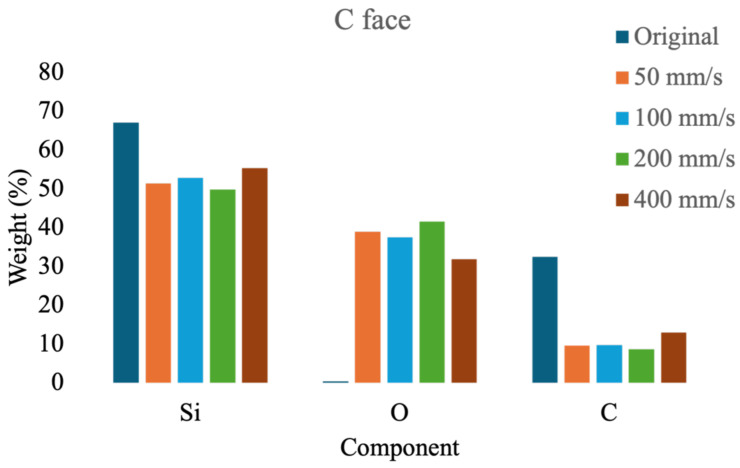
Ingredient analysis of the C face of SiC ablated with different scanning speeds.

First, compositional analysis was performed on both the Si and C faces of SiC before laser ablation and after ablation at varying speeds, as shown in Figure 8 and Figure 9. The experimental results reveal that the compositional ratios on both the Si and C faces are highly consistent before and after laser ablation. Initially, the Si and C average weights were 67.15% and 32.4%, respectively, with minor surface oxidation at 0.45%. Following laser ablation at a higher scan speed (400 mm/s), the average compositions of Si, O, and C on both surfaces were found to be 55.35%, 31.65%, and 13%, respectively. However, when the scan speed decreased to below 200 mm/s, oxidation increased by approximately 8–10%, leading to Si content stabilizing between 48 and53% and C content between 8 and10%. The findings indicate that when the scanning speed drops below 200 mm/s, the oxidation degree of the material will not change significantly as the scanning speed decreases.

**Figure 10:**
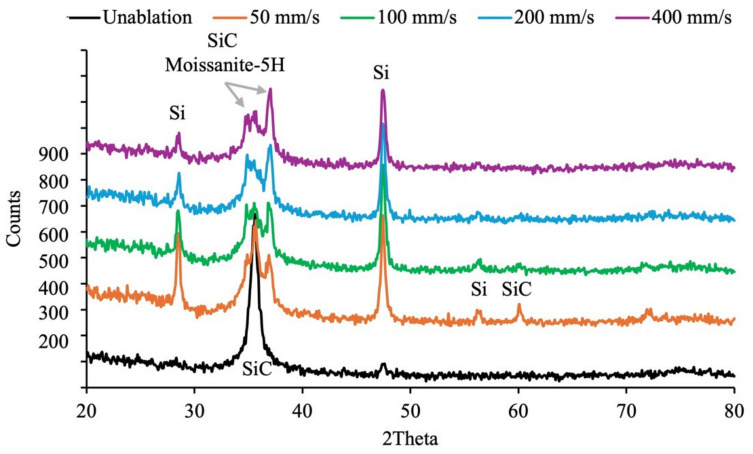
XRD diagram of the Si face of SiC before and after laser ablation with varying scanning speed.

**Figure 11:**
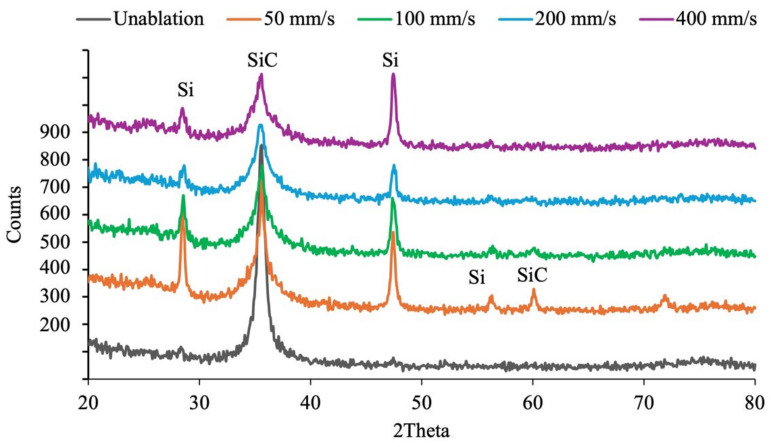
XRD diagram of the C face of SiC before and after laser ablation with varying scanning speed.

In terms of lattice structure analysis, both the Si and C faces of SiC exhibit the standard hexagonal lattice structure of SiC prior to laser treatment. However, after laser ablation, the Si face transforms into a structure that includes silicon (Si) in a cubic arrangement and Moissanite-5H SiC, as shown in Figure 10. Moissanite-5H is a unique polytype of silicon carbide that differs significantly from the more common polytypes, such as 4H-SiC, 6H-SiC, and 3C-SiC. It remains a hexagonal structure and has a unique stacking sequence, mainly in a hexagonal symmetry arrangement that is periodically repeated every five layers. On the other hand, laser ablation of the C face results primarily in a partial transformation of the lattice structure into silicon (Si), accompanied by a decrease in the original SiC response, as shown in Figure 11. No additional polytypes or structural forms appear on the C face. As the scanning speed decreases and the ablation effects intensify, the lattice structure on both sides of C and Si will be rearranged, and the response of SiC can be measured again.

**Figure 12:**
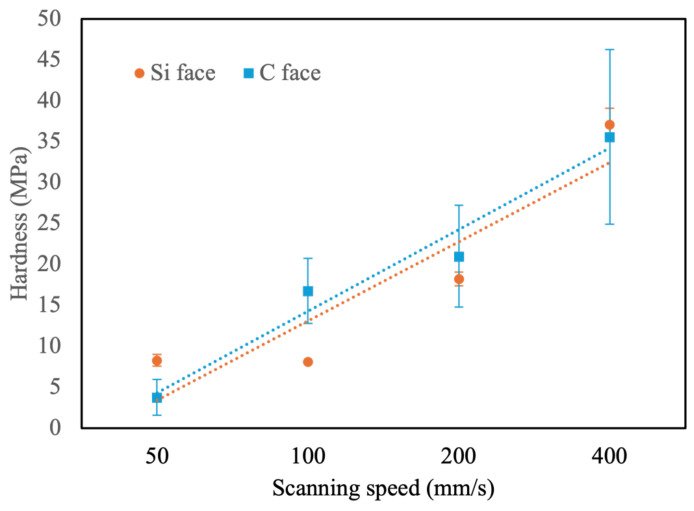
Hardness of SiC ablated with different scanning speeds.

The original hardness of the Si and C faces of SiC is notably high, as shown in Table 2, with the C face (13,896 MPa) being harder than the Si face (11,198 MPa). This disparity is due to variations in atomic bonding and crystal orientation. On the C face, the termination with carbon atoms allows for stronger C-C bonds compared to the Si-Si bonds on the Si face. Additionally, differences in crystal stacking structure contribute to the higher density and hardness of the C face. However, after laser ablation, the hardness of both faces dramatically decreases to less than 1% of their original values. The hardness has a linear relationship with the scanning speed. When the scanning speed decreases, the ablation effect is high, and the hardness will be relatively lower (as shown in Figure 12). At scanning speeds below 400 mm/s, the hardness on both faces falls below 100 MPa. This decrease in hardness has considerable advantages for the subsequent CMP process.

### 4.3. Influence of Repetitions on Surface Morphology and Properties of SiC

**Figure 13:**
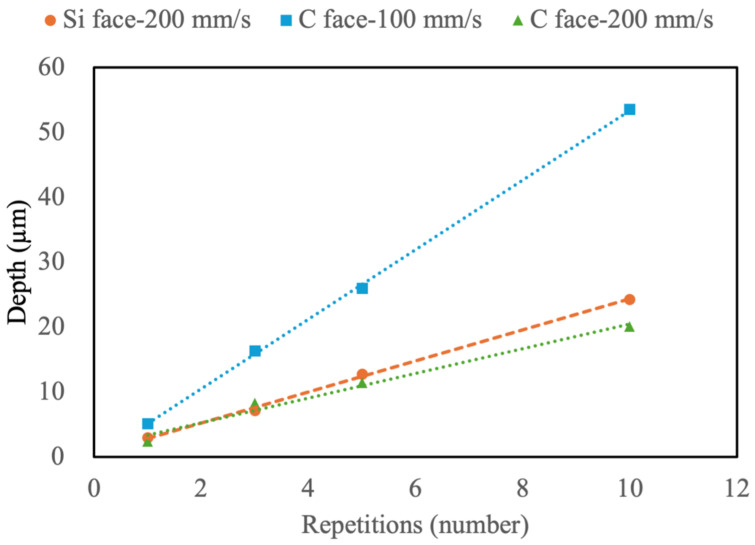
Depth of the Si face and C face ablated by different scanning speeds and repetitions.

**Figure 14:**
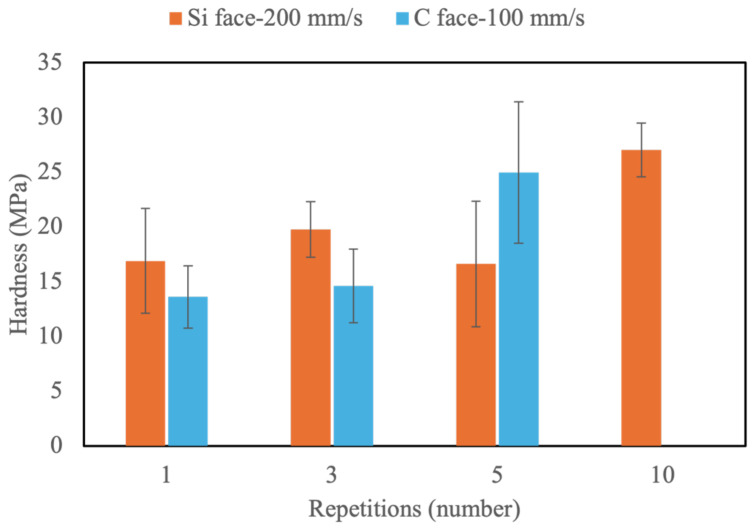
Hardness of the Si face and C face treated by different repetitions.

From the above experimental results, the hardness of SiC significantly decreases following laser ablation, primarily due to material oxidation and alterations in the internal lattice structure. In terms of ablation depth, a scanning speed of 200 mm/s yields the most effective results for the Si face, while for the C face, speeds between 100 and 200 mm/s demonstrate greater efficacy, with 100 mm/s outperforming 200 mm/s. Thus, further processing will be conducted at scanning speeds of 200 mm/s and 100 mm/s with varying repetitions to analyze the resulting ablation depth and surface hardness. As shown in Figure 13, the ablation depth exhibits a highly linear relationship with the number of laser repetitions. On the Si face, ten repetitions achieve an ablation depth of approximately 25 μm, whereas the same speed only removes around 20 μm on the C face. For optimal results on the C face, a scanning speed of 100 mm/s reaches a depth of 25 μm with five repetitions and 50 μm with ten repetitions, confirming that 100 mm/s with five passes achieves a 25% increase in efficiency over 200 mm/s with ten passes. Post-laser ablation, the hardness on both SiC faces consistently drops below 50 MPa after one to ten repetitions, as shown in Figure 14. Minor increases in hardness with repetition may result from surface irregularities induced by ablation.

## 5. Conclusions

This study systematically evaluates the effects of ultraviolet nanosecond laser ablation parameters on the surface morphology, composition, lattice structure transformation, and hardness of silicon carbide (SiC) across its Si and C faces. Results indicate that Si and C faces exhibit distinct behaviors under varying laser scanning speeds. Lower scanning speeds enhance ablation efficiency and induce more pronounced changes in lattice structure and composition. Notably, at speeds below 400 mm/s, surface roughness and material removal depth increase significantly, with optimal speeds of 200 mm/s for the Si face and 100 mm/s for the C face. At these reduced speeds, the C face surpasses the Si face in roughness, reflecting a higher sensitivity to ablation.

Additionally, laser-induced oxidation on the SiC surface raises the oxygen content by 30–40%, while structural changes include the formation of Moissanite-5H SiC and cubic silicon on the Si face, suggesting polymorphic transformations triggered by thermal and structural rearrangements. Conversely, the C face retains some crystalline SiC structure, though partial transformation to elemental silicon is observed. Post-ablation, hardness on both faces declines significantly, to less than 1% of the original value, which has substantial implications for subsequent chemical-mechanical polishing (CMP) processes. These findings emphasize that scanning speed and repetition times play a key role in silicon carbide laser processing and provide important reference information, including the need for precision control of processing parameters and material properties for the double-sided processing of advanced semiconductor materials such as SiC.

## Figures and Tables

**Table 1:** Specification of laser system and experimental parameters.

Parameter	Value
Wavelength (nm)	355
Laser power (W)	20
Pulse repetition frequency (kHz)	100
Y direction overlap rate (%)	50
Scanning speed (mm/s)	50, 100, 200, 400, 600, 800
Repetitions (number)	1, 3, 5, 10

**Table 2:** Hardness of the Si face and C face of SiC before laser ablation.

Hardness	Si Face	C Face
Mean (MPa)	11,198	13,896

## Data Availability

The original contributions presented in this study are included in the article. Further inquiries can be directed to the corresponding author.
